# The Role of Phytocompounds and the Physiological Response of the Skin in Common Dermatological Conditions: A Narrative Review and Bibliometric Analysis of Trends

**DOI:** 10.3390/ph19050757

**Published:** 2026-05-12

**Authors:** Csaba Nagy, Florina Miere (Groza), Mariana Ganea, Laura Grațiela Vicaș, Mariana Eugenia Mureșan, Angela Antonescu, Simona Ioana Vicas, Luciana Dobjanschi

**Affiliations:** 1 Doctoral School of Biomedical Science, University of Oradea, 1st University St., 410073 Oradea, Romania; nagy.csaba@student.uoradea.ro; 2 Preclinical Department, Faculty of Medicine and Pharmacy, University of Oradea, 10 Sq. 1st December Street, 410073 Oradea, Romania; mmuresan@uoradea.ro (M.E.M.); aantonescu@uoradea.ro (A.A.); dobjanschil@uoradea.ro (L.D.); 3 Department of Pharmacy, Faculty of Medicine and Pharmacy, University of Oradea, 1st December Street, 410073 Oradea, Romania; mganea@uoradea.ro (M.G.); lvicas@uoradea.ro (L.G.V.); 4 Faculty of Environmental Protection, University of Oradea, 26 Gen. Magheru Street, 410048 Oradea, Romania; svicas@uoradea.ro

**Keywords:** bibliometric analyses, dermal disease, phytocompounds, controlled system delivery

## Abstract

**Background**: The skin, as the largest organ of the human body, plays a crucial role in protection, immunity and homeostasis. Its exposure to environmental and internal factors contributes to the development of various dermatological conditions. Conventional treatments are often associated with adverse effects and increased resistance. This review aims to explore the growing role of phytotherapeutic approaches in dermatology, along with mapping recent research trends in the field. **Methods**: The paper presents three parts: the first part highlights the mapping of interest in the addressed topic through a systematic selection of the specialized literature using the Web of Science database. A bibliometric analysis was conducted using the Web of Science Core Collection, with data visualized in VOSviewer to identify publication trends, keyword clusters, and collaboration networks across European countries. Subsequently, in the second part of the review, the main topical topics regarding the skin were addressed (the immune and non-immune response system, microbiome composition and physiological responses in different situations). The third part of the paper addresses phytotherapy targeted at the dermatological sphere and controlled release therapeutic systems. **Results**: The analysis identified a total of 267 publications, with a significant increase in recent years. Key research clusters included phytochemical-based therapies, nanocarrier systems, and inflammatory skin conditions. Keyword co-occurrence analysis revealed emerging trends in nanoformulations and targeted delivery systems. The main research groups focused on polyphenols, antioxidant activity, anti-inflammatory effects and advanced delivery systems, such as nanoparticles and liposomes. In addition, innovative formulations have improved bioavailability and targeted administration. **Conclusions**: Phytotherapeutic approaches represent a promising alternative to conventional dermatological treatments, offering effective, safer and more sustainable solutions. The integration of natural compounds with modern delivery systems improves therapeutic outcomes and minimizes side effects, supporting their increasing relevance in clinical and pharmaceutical research.

## 1. Introduction

The skin is the largest organ of the human body and the area of direct contact with the environment [1]. It can therefore be said that the health of the skin directly influences the health of the human body because it is the first barrier of the human body and has a defining role in the body’s defense process [2].

The skin is a complex organ made up of three layers (epidermis, dermis and hypodermis) but especially of its own, complex immune system that allows the self-regulation of its homeostasis and functions as the first line of defense of the human body [1,3,4].

From a microbiological point of view, the skin supports a complex microbiome, made up of bacteria, fungi and viruses that have the role of functioning together with existing cells as an independent immunological organism [5].

All these physiological aspects, or the health of the skin, can be influenced by several internal factors of the body or external environmental factors, so that throughout life, this organ can undergo multiple changes [6,7].

The main external factor that influences the health of the skin is exposure to UV or VIS radiation, with an increased risk of inflammation, superficial wounds or, through prolonged exposure, major wounds or burns. Derived from these, pathologies (often frequent) such as those characterized by inflammation (including wounds and microbiological infections), depigmentation or hyperpigmentation can exist throughout life. Also, the appearance of diseases that have oxidative stress and increased levels of free radicals such as skin cancer as their starting point can be encountered when physiological, immunological and microbiological defense mechanisms are destabilized [8,9].

On the other hand, skin diseases represent a significant global health burden, affecting millions of individuals worldwide and often requiring complex therapeutic approaches [10]. According to the World Health Organization (WHO), skin diseases remain among the most prevalent non-fatal conditions globally, emphasizing the need for innovative therapeutic strategies [10].

All of these conditions mentioned are conventionally treated with synthetic medicinal substances, such as formulas based on compounds with anti-inflammatory, corticosteroid, and antimicrobial properties, etc. [11].

The major disadvantage of these conventional treatments is that over time, they offer unwanted side effects but more than that, the so-called resistance to the active substance may develop, with the therapeutic effect being greatly attenuated or sometimes non-existent [12,13].

For this reason, the trend in recent years has been toward the development of innovative pharmaceutical formulas that contain phytocompounds with an essential role in treating various dermatological conditions. Thus, the appearance of side effects is avoided, but above all, the existence of resistance to treatment is avoided [14].

At the same time, the trend is to incorporate phytocompounds extracted from plants that are proven to have a concrete therapeutic effect, in therapeutic delivery systems such as nanoemulsions, emulgels and nanocarriers such as liposomes [15].

These therapeutic systems make the treatment of skin conditions more efficient because they present advantages such as targeted and sustained release of phytocompounds in the target area, maintaining their high bioavailability and minimizing possible side effects [16].

This synthesis paper aims to highlight the topicality and relevance of the subject of phytocompound-based skin therapy.

The paper is innovative in that it addresses three distinct directions that intersect at a common point: the increased interest in the use of phytocompounds as an alternative treatment for skin conditions, thus managing to increase their compliance and bioavailability compared with conventional treatments.

Unlike previous studies, this work integrates bibliometric analysis with a structured narrative review, providing a comprehensive perspective on research trends and the therapeutic potential of phytocompounds in dermatological applications.

Initially, through a bibliometric study conducted over a five-year period (2021–2026), the article highlighted the publication trends, the most popular study centers and the situation of the subject addressed in continental Europe, emphasizing that Romania shows increased interest along with other European countries for the subject addressed. Subsequently, the paper continues to focus on the previously mentioned subjects, including current studies on the topic addressed, not only those presented in the bibliometric analysis. Thus, as a distinct point, the paper highlights important notions about the skin microbiome and its physiology, the immune and non-immune response systems, in order to subsequently facilitate the understanding of the main notions regarding the most common dermatological pathologies.

As mentioned, the increased trend of including treatments based on compounds derived from plant extracts and their inclusion in controlled-release systems is a third point reached in this paper. The main classes of phytocompounds and controlled-release systems that allow an increase in the efficiency of dermatological treatment compared with classic, conventional formulas, are thus presented.

Therefore, the purpose of this analysis is to elucidate the physiological mechanisms of skin defense and repair, the most common situations in the pathological sphere of this field and the presentation of new treatment alternatives based on natural compounds.

## 2. Bibliometric Study, Exploratory and Descriptive Research Design

### 2.1. Screening of Scientific Studies with Research Topics Common to the Field of Interest in Order to Carry Out This Scientific Review

Our study was conducted as an exploratory and descriptive research paper on the topic of maintaining skin health with the help of phytochemical compounds present in various plant extracts.

**Figure 1:**
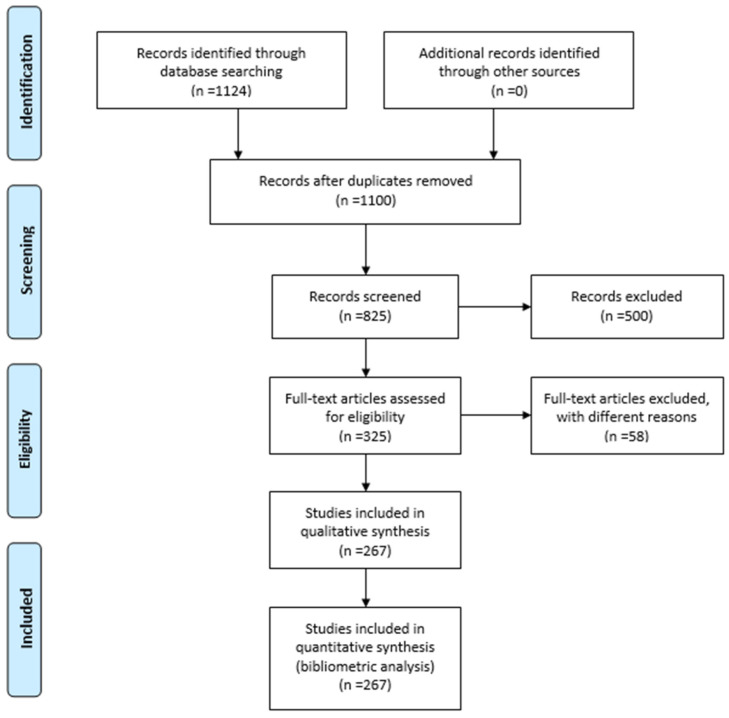
Flow chart of the PRISMA 2020 study selection process [17].

Figure 1 shows the flow chart of the PRISMA 2020 study selection process [17]. The literature search was performed in the Web of Science Core Collection database using title and abstract filters related to phytocompounds, phytotherapy, and dermatological conditions, described further in the bibliometric analysis below. Initially, 1124 records were identified, while no additional studies were retrieved from other sources. After removing duplicates, 1100 records remained for screening. Following the evaluation of titles and abstracts, 500 records were excluded because they were not sufficiently relevant to the research topic. Subsequently, 325 full-text articles were assessed for eligibility, and 58 studies were excluded due to insufficient data, lack of dermatological relevance, or absence of investigations focused on phytocompounds. To highlight the European research landscape and collaborations involving Romania, an additional geographical selection criterion was applied, retaining only studies published by authors affiliated with European countries. As a result, 267 studies were finally included in the qualitative synthesis and quantitative bibliometric analysis.

To increase the accuracy of the approach to the chosen topic, bibliometric techniques were used to identify trends in the field of interest in terms of research and specialized literature. Bibliometric analysis allows for a broad view of recent research and the creation of clusters (links and interconnections) between studies, facilitating the presentation in a review article of the latest discoveries and trends in the topic of interest.

In other words, the results obtained with the help of bibliometric analysis allow for an integrated picture of the current state of knowledge, including the identification of innovations and scientific trends, as well as contributions from prolific authors, institutions, countries, and high-impact journals.

The keywords used in this bibliometric analysis were chosen to accurately reflect the major research directions regarding the maintenance of skin health, the main pathologies present at this level and the treatment alternatives based on phytocompounds derived from extracts of the various plants included in the respective studies and their way of intervening in the preventive or healing process of the epidermis.

### 2.2. Search Strategies

The search of the specialized literature was carried out using the Web of Science (WOS) database (Clarivate, 12 January 2026), including the Science Citation Index Expanded (SCI-EXPANDED), Emerging Sources Citation Index (ESCI), Conference Proceedings Citation Index-Science (CPCI-S) and Book Citation Index-Science (BKCI-S) databases. The articles selected for research were published within the period 1 January 2021–1 January 2026, with these representing the research carried out in the phytotherapeutic field with skin applications over the last 5 years.

Bibliometric data retrieved from the Web of Science Core Collection were analyzed and visualized using VOSviewer to quantify co-authorship links, map keyword co-occurrence, identify the most frequent keywords, and assess country-level publication output across European countries. The search strategy was defined using the following query: TI = (“phytochemical” OR “polyphenol”) OR AB = (“dermal disease*” OR “flavonoid*”) AND (AB = (antioxidant* OR immunomodulator* OR anti-inflammatory OR antiviral* OR anticancer*)).

The process of selecting relevant articles was optimized by applying search filters exclusively at the title (TI–Title) and abstract (AB–Abstract) levels. This strategy was adopted to ensure the strict inclusion of studies in which the investigated concepts constitute the central core of the research, thus minimizing the risk of retaining peripheral works or with low relevance to the study objectives. It was decided to abandon the topic search filter (TS–Topic), because it was found that it generates a high number of false-positive results. This data distortion was mainly caused by the automatic inclusion of Keywords Plus terms in the Web of Science platform. These terms, being derived from the titles of cited references and not necessarily from the actual content of the article, introduced erroneous associations. By restricting the search to the TI and AB fields, articles where the terminology of interest appeared only in metadata or in adjacent contexts without an intrinsic link to the study topic were systematically excluded. This methodological approach guaranteed a high precision of the dataset, reflecting the real and direct correlation between the targeted phytocompounds (polyphenols, fatty acids, flavonoids) and their biological effects at the cutaneous level.

### 2.3. Bibliometric Analysis of Keywords

The bibliometric analysis was performed using the VOSviewer software, version 1.6.20.

As previously mentioned, the time interval considered was 5 years, so by using the mentioned keywords and selecting the years 2021–2026, 267 relevant articles related to the abstract topic were identified.

Also, in addition to the time period, keywords and the top 10 journals that published in the field of interest were selected (Mdpi, Elsevier, Wiley, Frontiers Media Sa, Springer Nature, Nature Portfolio, Taylor & Francis, Massachusetts Medical Soc., Amer Assoc Advancement Science). The articles selected and studied are “article” or “review” type articles, the most cited of the total number of articles identified.

**Figure 2:**
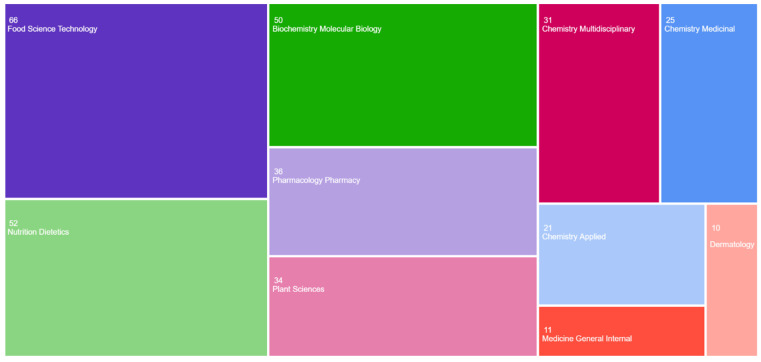
TreeMapChart representation of the topic of publications in the field of interest (plant extracts or compounds with an effect on dermis health) and the number of publications on each topic during the period 1 January 2021–1 January 2026.

Also, only European countries were selected, in order to evaluate the degree of interest for the topic addressed in the European area (including Romania). The selected articles were published in journals that address topics of interest for our study, which are presented in Figure 2. It can be seen that the areas of interest for addressing strategies to maintain the health of the human body and implicitly of the skin are “food science technology” or “Nutrition Dietetics”, but most of the published articles provide information on the molecular biochemistry and pharmacology of plant extracted compounds. Relevant notions regarding the topic debated in this review were also identified in areas such as dermatology or multidisciplinary sciences. In other words, the multitude of topics used on the topic addressed highlights a constant and sustained trend of interest towards potential phytocompounds with applicability in common dermal diseases (lesions, acne, photoprotection, etc.).

The articles selected to address our topic of interest in the most complex way were also saved as a database and characterized using the VOSviewer software, version 1.6.20.

**Figure 3:**
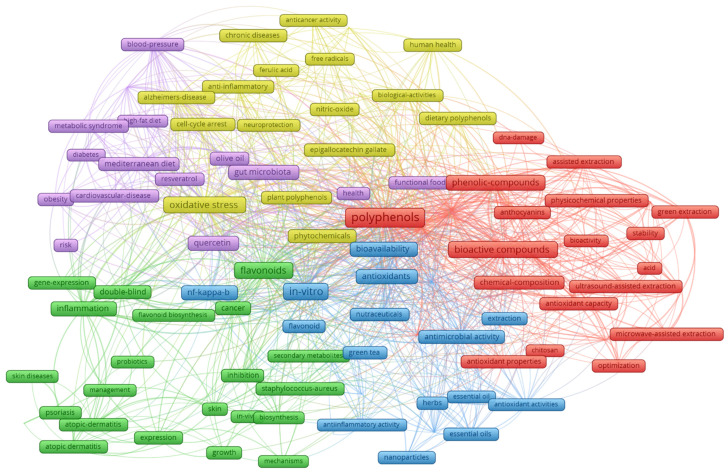
Co-occurrence network of the keywords used and their interdependence with other adjacent words on the same topic.

Initially, the keywords used were taken into account, and thus the co-occurrence network between them was generated (Figure 3).

The VOSviewer map shows five main research clusters (red, green, blue, purple and yellow) related to polyphenols, indicating interconnected areas of study.

Red cluster: This cluster focuses on methods for obtaining bioactive compounds, including green extraction, microwave/ultrasound-assisted extraction and physicochemical properties. Key terms include “phenolic compounds”, “bioactive compounds” and “antioxidants”.

Green cluster: The largest cluster, exploring the diverse therapeutic effects of polyphenols in the prevention and management of chronic diseases, especially cancer and inflammatory (skin) diseases. Important terms are “anticancer activity”, “atopic dermatitis”, “psoriasis”, “NF-kappa b” (an inflammation signaling pathway) and other dermatological diseases.

Blue cluster: This cluster details the mechanisms of action and the specific compounds responsible for the pharmacological effect. Topics include “oxidative stress”, “bioavailability” and the role of polyphenols in dermal diseases. Advanced delivery systems such as “nanoparticles” are also mentioned as methods to improve the efficacy of the compounds.

Purple cluster: This is a general cluster that focuses on the connection between polyphenols, human health and dietary sources. Terms include “human health”, “Mediterranean diet”, “blood pressure” and neurodegenerative diseases such as Alzheimer’s disease.

Yellow cluster: This focuses on specific phenolic compounds and their connection to various aspects of human health, chronic diseases and diets. Key elements of the yellow cluster include ferulic acid (ferulic acid) and gallic acid (gallic acid), which are central, highlighting research on individual compounds with specific properties. The terms “human health” and “chronic diseases” indicate that this cluster links these specific compounds directly to their impact on general health and disease management. The links with the “Mediterranean diet” and “plant polyphenols” emphasize the natural sources and nutritional importance of these compounds. Various biological and anti-inflammatory activities are mentioned, suggesting an exploration of how these compounds interact with the body at the molecular level.

Therefore, each cluster represents a distinct but closely related field of research, demonstrating the interdisciplinary nature of studies on polyphenols.

**Figure 4:**
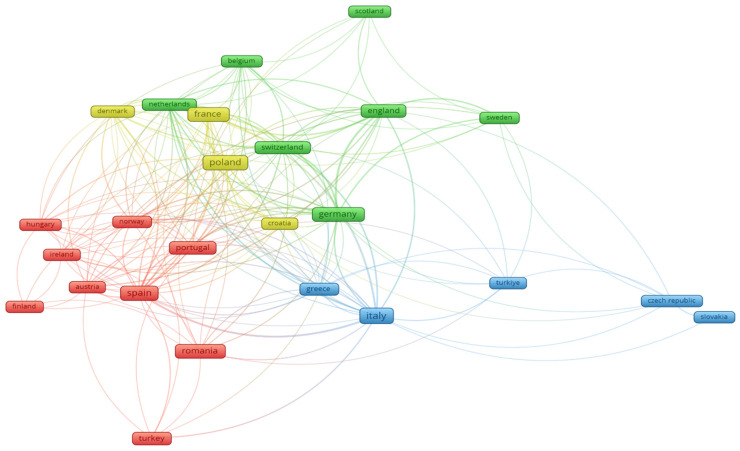
Map of the European Interconnection Network and interest in the same research topics (phytocompounds and mechanisms of action on skin pathologies).

When selecting the articles of interest for this review, filters were imposed in order to consider only articles published by authors affiliated with institutions in Europe. This selection was made to highlight the existing collaborations between Romania and European countries. Thus, Figure 4 represents and highlights the main working groups (interconnected countries) that research the topic of interest addressed in this review (phytocompounds, their mechanisms of action on the skin and their use in the healing process of dermatological diseases).

The map generated with the VOSviewer software presents a network of international collaboration or research on the same topics of interest and is structured into four main clusters (red, green, blue and yellow).

The red cluster (Central and Northern Europe) includes countries such as Romania, Finland, Austria, Hungary, Ireland, Spain, Norway and Turkey.

The green cluster (Western and Northern Europe) represents a strongly interconnected network that includes the United Kingdom (England, Scotland), Belgium, Switzerland and Sweden.

The blue cluster (Southern and Eastern Europe) includes Italy, Greece, Turkey, the Czech Republic and Poland.

The yellow cluster (Eastern Europe and France) includes Poland, France, Romania, Portugal and Denmark.

Romania’s connections with Europe are highlighted through links between the red cluster and the other clusters, especially with countries such as Italy, Spain, Greece, Turkey, Poland, Germany and France.

Romania is part of the red cluster, indicating close collaboration with countries in this group or common research topics with them and beyond.

The focus on Europe, particularly Romania, reflects the regional research landscape and institutional collaboration patterns that are relevant to the authors’ academic context. This approach allows for a more detailed analysis, rather than a broad but less specific global overview.

This map highlights that Romania has a diverse collaboration network, with strong ties within its core group and less intense, but existing, connections with other European regions.

## 3. Skin Physiology, Microbiome Composition and Physiological Response Capacity Against Disruptive Factors

The skin, or the largest organ of the human body, can reach a surface area of between 1.5 and 2 m^2^ and represents, as has been presented since ancient times, the barrier or delimitation of the body from external factors [18].

However, the epithelial system is much more complex than this simple description, taking into account the complexity of the composition but also the characteristic immune response capacity [19].

Thus, the skin is made up of three layers—the epidermal layer, the dermis and the hypodermis—but in addition to these, the skin appendages (hair follicles and sebaceous and sweat glands) are present, and last but not least are the physiological microbiome and immune cells that are capable of forming the so-called lymphoid immune response system [20].

The epidermis is the outer layer of the skin and is in direct contact with the environment, being made up of up to 90–95% of keratinocyte-type cells. From the outside of the skin (the area of contact with the external environment), the epidermis is made up of four distinct areas such as: the stratum corneum (represented by dead keratinocytes), the stratum lucidum, the stratum granulosum and the basal layer [9].

The dermis is found immediately below the epidermal layer, in direct contact with its basal layer, and at this level are immune cells such as macrophages, mastocytes, lymphocytes, which are included in the most recent scientific works. These are found together with fibroblast cells, and an extracellular matrix of collagen and elastin, together forming a very well-structured network that allows the “mobilization” of immunological cells to have an immediate and firm immune response to disturbing factors [21].

The hypodermis is rich in adipocytes, fibrocytes and blood vessels, being the deepest layer of the skin. At this level, the immune response also takes place by stimulating the production of cytokines and manages to maintain skin heat homeostasis through the adipokines formed (fat being practically low in heat conductivity, protecting its loss) [4].

The skin microbiome plays a major role in maintaining homeostasis and health. The wide variety of microorganisms that make up the skin microbiome include: bacteria, viruses and some fungi. The composition of the dermal bacterial flora is different depending on the area considered for research. Four areas can be distinguished: dry skin area (most of the skin), skin area rich in sweat glands, area with sebaceous glands (especially the face area), and the sole of the foot area [3].

**Figure 5:**
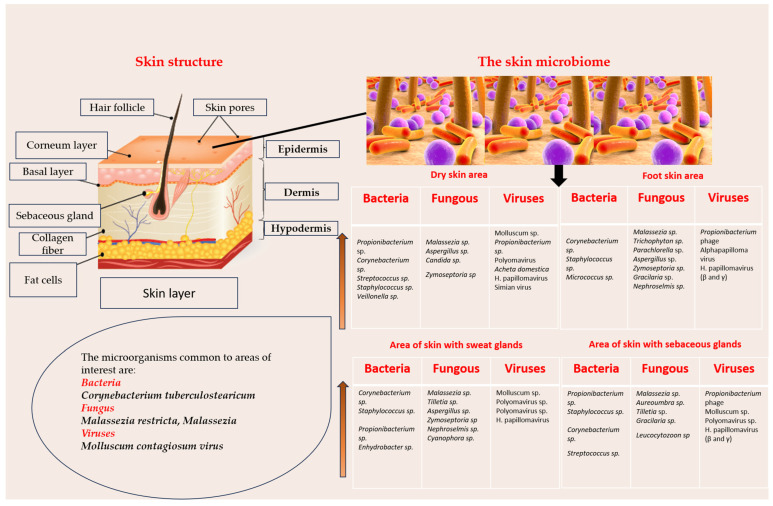
Physiological structure of the skin and composition of the skin microbiome, depending on the areas studied. The orange arrows in gradient (from light orange to dark orange) suggest the quantitative presence of the bacteria, fungi or viruses noted in the image, depending on the area.

The top of the microorganisms was researched and identified depending on the skin area, with the results being presented in Figure 5.

The highest frequency was observed for the microorganisms indicated by the arrows. According to Figure 5, the bacteria found on all four skin areas (including areas with abundant sebaceous glands or areas with abundant sweat glands) is *Corynebacterium tuberculostearicum*. Also, the bacteria most frequently found in three of the four areas under consideration are: *Staphylococcus hominis*, *Staphylococcus epidermidis*, *Propionibacterium acnes*, *Micrococcus luteus*, *Corynebacterium simulans* and *Staphylococcus capitis*. For example, *Staphylococcus hominis* and *Staphylococcus epidermidis* are present on the skin of the legs, in areas with sebaceous and sweat glands, but *Propionibacterium acnes* are characteristic of dry skin but also in areas with sebaceous and sweat glands. *Micrococcus luteus* was not identified in the area with sweat glands [3].

The skin microbiota includes fungal species such as *Malassezia restricta* and *Malassezia globosa*, as well as viruses like the *Molluscum contagiosum* virus, commonly identified across different skin regions [22]. The composition of the skin microbiome is dynamic and varies with age. In early life, it is influenced by the mode of delivery and close environmental exposure, while during puberty it undergoes significant changes driven by hormonal and biochemical factors, often associated with inflammatory skin conditions [3,23,24].

The skin immune system, located primarily in the dermis, consists of dendritic cells, macrophages, T lymphocytes, and innate lymphoid cells, forming skin-associated lymphoid tissue (SALT) [9,24,25]. The myeloid lineage includes Langerhans cells, dermal dendritic cells, macrophages, mast cells, and eosinophils, while the lymphoid lineage comprises T cells and natural killer T cells [3,9,26,27].

In addition to immune cells, structural cells such as keratinocytes, melanocytes, and adipocytes actively contribute to immune responses. These cells can recognize antigens, secrete proinflammatory cytokines (e.g., IL-1, IL-6, TNF-α), and interact with immune cells [4,19,27]. Overall, the skin functions as a complex immunological organ, extending beyond its role as a physical barrier [28,29,30,31].

## 4. The Most Common Skin Conditions

### 4.1. Skin Photoaging

The skin’s aging processes are due to two essential factors: the first factor is genetic or chronological aging of the skin and the second factor is the external factor or exposure to UV light [32].

The second factor causes an acceleration of skin aging due to cell destruction produced at the epidermis level and within the deeper layers [32,33].

UV light is the most harmful external factor that causes aging or disease (especially after with prolonged exposure) of the skin. UV light is made up of UVA, UVB and UVC light, thus covering different wavelengths, and therefore a wide absorption spectrum: UVA (ƛ = 320–400 nm), UVB (ƛ = 280–320 nm) and UVC (ƛ = 100–280 nm). Of these, UVA and UVB reach the skin in the event of exposure, becoming harmful to skin health over time. UVC radiation being absorbed at the ozone layer level is not considered harmful to the health and integrity of the skin [32,34].

Through the innate immune system and through its stratified structure and composition consisting of in different types of cells, the skin is able to “defend” itself against UVA and UVB, although this defense capacity is limited and influenced by the time of exposure. With prolonged exposure, however, UVA radiation acts on the skin in a destructive way by forming free radicals and producing oxidative stress or by modulating enzymes before altering, improving the immunological response to other external factors. UVB radiation, however, initially acts at the superficial level in the epidermis and subsequently penetrates deeper into the skin [20,32,35]. Regardless of the type of exposure to UVA or UVB radiation, the following can occur at the dermis level: accelerated photoaging, burns, photo-dermatosis, hyperpigmentation, precancerous or cancerous lesions (depending on the degree of DNA destruction) [36]. UV radiation induces a process of photosensitization of endogenous biomolecules (such as melanin, NADH and urocanic acid), a phenomenon that catalyzes the generation of reactive oxygen species (ROS), with direct cytotoxic effects on the integrity of lipid membranes, protein structures and genetic material. This redox imbalance activates surface receptors (EGF, KGF, IL-1, TNF-α) and intracellular signaling cascades (AP-1, NF-κB), leading to a systemic pro-inflammatory response marked by the release of lipid mediators, such as prostaglandin-F2α and 12-HETE, through the up-regulation of cyclooxygenase (COX) and lipoxygenase enzymes. In fibroblasts, UVA/UVB-induced oxidative stress inhibits proteasomal activity and stimulates autophagy as a survival mechanism, processes that finalize the cellular senescence phenotype, while keratinocytes can exhibit adaptive responses by modulating the polyunsaturated fatty acid (PUFA) profile, such as increasing the concentration of docosahexaenoic acid (DHA), to facilitate post-exposure regeneration [7,29,37].

### 4.2. Skin Inflammation

Skin inflammation can be physiological in nature or can be caused by or associated with other symptoms in various dermatological conditions.

From the point of view of the time of appearance and healing of physiological inflammation, this can be acute physiological inflammation or chronic physiological inflammation. Regardless of the situation, the body’s defense mechanism is approximately the same and is characterized by a cascade of cellular and immunological reactions [38].

**Figure 6:**
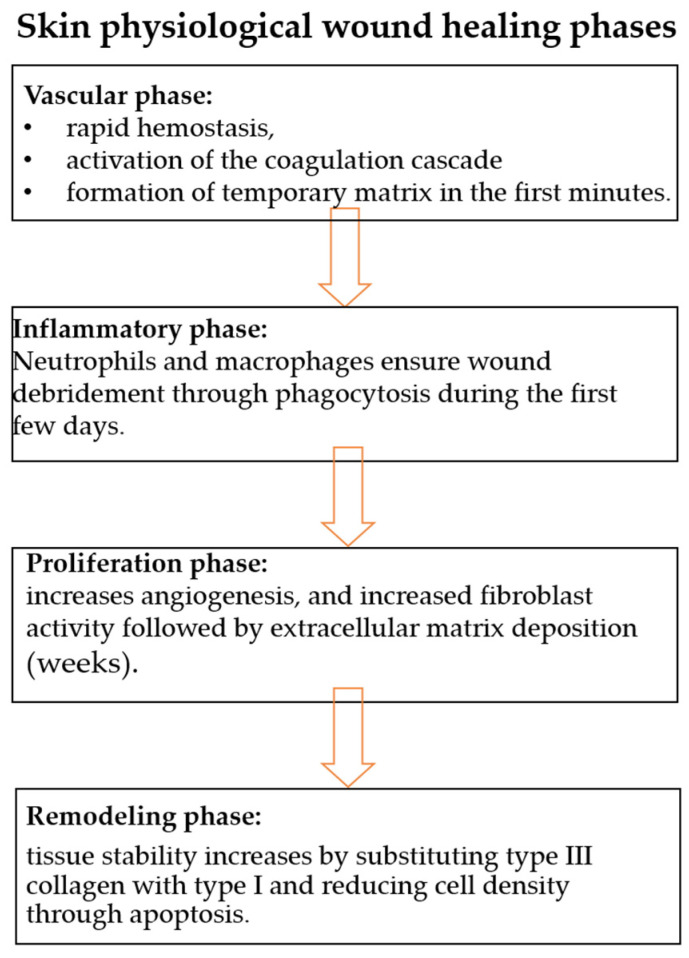
The stages of healing and scarring of a wound.

In the case of acute inflammation, in the first phase, redness, heat, swelling and pain appear at the level of the skin lesion (a reaction that is actually controlled by the innate dermal immune system) (Figure 6). From a biochemical point of view, nociceptors in the skin come into action through the secretion of neuropeptides, which have a vasodilator and proinflammatory effect. In other words, there is a feedback relationship between dermal cells and nerve cells that is greatly intensified in the case of lesions that cause acute inflammation [39,40].

Deep down, pro-inflammatory reactions are intensified by the degranulation of mast cells and thus by the release of biochemical compounds such as histamine and prostaglandins. These dictate chain reactions that favor vasodilation in the deep skin area; thus, the final effect is a pro-inflammatory one [41,42]. This stage is in fact the first path towards healing and regeneration of the inflamed tissue. In order for the healing process to be achieved, the stage of increasing neutrophil adherence and chemotaxis is necessary; thus, intercellular adhesion and the release of vascular endothelial growth factor will in turn determine local angiogenesis. At the site of the inflammatory lesion, interleukin IL-8, C5a and LTB4 are mobilized, which will decrease the inflammation and, together with neutrophils (with phagocytose bacteria present on the skin at the time of the inflammatory lesion) will prevent its eventual infection and chronicity [43,44,45].

Pathological inflammation can occur when one of the physiological repair processes of a physiological is disturbed. Pathological inflammation generally occurs in chronic dermatological conditions such as: contact dermatitis, psoriasis, rosacea, hidradenitis suppurativa, necrosis and acute or infected wounds [40,43].

### 4.3. Skin Hyperpigmentation and Hypopigmentation

Hyperpigmentation is determined by the intensification of the melanogenesis process, a pathophysiological process mediated by a multitude of endogenous and exogenous factors. The main identified determinants include ultraviolet (UV) radiation, specific dermatological conditions, endocrine fluctuations, the senescence process, genetic predisposition, and cutaneous trauma or inflammatory processes [46,47].

#### 4.3.1. Influence of UV Radiation and Hormonal Factors

Exposure to solar radiation is the most significant etiological factor, acting as a major physiological stimulus on melanin synthesis [48]. Recent research highlights that early and cumulative exposure to UV radiation accelerates the worsening of dyschromia, causing clinical manifestations similar to melasma, post-inflammatory hyperpigmentation and senile lentigines [49,50,51]. At the endocrine level, female sex hormones—especially estrogen and progesterone—enhance the sensitivity of melanocytes to solar radiation. This mechanism explains the increased prevalence of chloasma and melasma among the female population, with these conditions being frequently correlated with gestational age or with the administration of hormone replacement therapies [52,53].

#### 4.3.2. Physiological Changes Associated with Senescence and Genetics

The aging process induces structural changes at the level of the melanic unit; although the total number of melanocytes progressively decreases, the residual cellular units show phenomena of hypertrophy and functional specialization [54,55,56]. These physiological alterations become clinically manifested, usually, after the fourth decade of life. Complementarily, the pigment architecture is governed genetically, with the profile of gene polymorphism being the one that regulates the differentiation and functionality of melanocytes, thus determining the cutaneous phenotype [36].

#### 4.3.3. Post-Inflammatory Hyperpigmentation (PIH) and Acne Pathology

Post-inflammatory hyperpigmentation occurs as a sequela of inflammatory processes or traumatic lesions, including exposure to chemical agents, thermal burns, wounds, or chronic conditions such as psoriasis, atopic dermatitis and acne [57].

From a pathophysiological point of view, deep dermal inflammation—manifested by papules and pustules—can induce an aberrant synthesis of melanin. In the case of acne, it has been observed that mild forms present a low risk of dyschromia, but mechanical traumatization of the lesions (compression, penetration) exacerbates the melanocytic response, leading to the formation of persistent pigment deposits [58,59].

#### 4.3.4. Iatrogenic Factors and Systemic Correlations

The etiological spectrum of hyperpigmentation also includes iatrogenic factors, such as the administration of certain pharmacological classes: antibiotics, oral contraceptives, antimalarials and tricyclic antidepressants [58,60,61]. At the same time, hyperpigmentation can occur as a secondary adverse effect of phototherapy procedures or laser treatments. In rare cases, pigmentary changes can be a clinical indicator of systemic pathologies, such as Addison’s disease, characterized by hypofunction of the adrenal glands [62].

Hypopigmentation, characterized by a reduced concentration of melanin, has previous skin trauma as its main etiological cause [63,64]. Traumatic lesions, such as thermal or chemical burns, blisters, bacterial infections and other solutions for continuity of the skin tissue, can alter the integrity of melanocytes [65,66]. The reepithelialization process after such traumas often results in a skin area with a paler shade compared to the adjacent healthy tissue [67,68].

Depigmentation is the total loss of melanin pigment, leaving the skin a chalky white. Vitiligo is the reference example for this category, being an autoimmune condition characterized by the selective destruction of melanocytes. Clinical manifestations consist of well-demarcated white macules that tend to coalesce into large plaques. Although it is sometimes mistakenly perceived as a minor condition, its impact on skin homeostasis and the patient’s quality of life is significant [65,69].

### 4.4. Skin Wounds with Possible Microbial Infection

The existence of skin lesions throughout life is inevitable. In the healing process, physiological factors from the dermis are included so that the reepithelialization process is carried out gradually.

The healing process of skin wounds is a complex and sequential biological mechanism, which evolves from the vascular phase (characterized by rapid hemostasis, activation of the coagulation cascade and formation of a temporary matrix in the first minutes), to the inflammatory phase, where the recruitment of neutrophils and macrophages ensures the debridement of the wound by phagocytosis during the first days [70,71]. This stage is followed by the proliferation phase (days–weeks), defined by angiogenesis, and the increase in fibroblast activity followed by the deposition of the extracellular matrix, with the process culminating in the remodeling phase (months–years), in which tissue stability increases by substituting type III collagen with type I and reducing cell density through apoptosis, with the final result being an avascular and acellular scar tissue [72,73]. When these processes are interrupted by the existence of certain microbial agents, microbial skin infections occur, which can subsequently be accompanied by complications in existing wounds [74,75].

**Figure 7:**
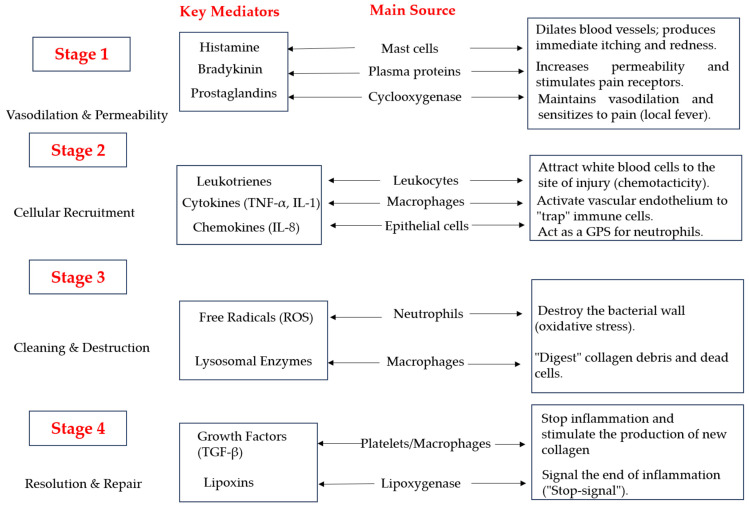
Common skin pathologies that are frequently encountered and the physiological mechanism of action that is characteristic of the healing process.

Figure 7 highlights common, frequently encountered pathological situations and the physiological mechanism of action that is characteristic of the skin healing process.

## 5. Phytochemical Compounds Recognized for Their Beneficial Properties on the Skin and Their Mechanisms in the Healing Process of Dermal Pathologies

Conventional treatments applied to the skin since ancient times have lost ground today due to the emergence of the phenomenon of resistance and decreased compliance with treatment. In order to treat or prevent various skin pathologies, attention is currently being directed towards natural compounds extracted from plant sources, which can be applied in different pharmaceutical forms (classical or innovative) to the skin so that treatment compliance is increased, but especially so that the response to treatment is fast, prompt and without the development of adverse reactions (often encountered in the case of classic dermatological treatments) [12,76].

Therefore, from the category of phytocompounds recognized for the healing properties of skin conditions, specific classes can be mentioned such as: polyphenols (along with their subclasses such as: flavonoids, flavanols, isoflavones, chalcones, anthocyanins, proanthocyanidins and nonflavonoids such as phenolic acids, stilbenes, etc.), alkaloids, polysaccharides, and peptides [12,77,78].

The mechanisms of action of these phytocompounds are extremely diverse and complex, depending on the existing pathology at the skin level, but all these phytocompounds act at the molecular level by initially reducing the stress caused by the existence of free radicals.

Several studies conducted to demonstrate the therapeutic capacity of various phytocompounds and plant extracts and to determine their characteristic mechanisms of action are presented in Table 1 and Table 2.

Table 1 and Table 2 summarize the main phytocompounds and plant-derived extracts investigated for dermatological applications, together with their experimental models, administration routes, biological activities, and proposed mechanisms of action. Most studies reported significant antioxidant, anti-inflammatory, wound-healing, photoprotective, antifungal, and anti-aging properties. The investigated materials were evaluated through both *in vitro* and *in vivo* models, including keratinocyte and fibroblast cell lines, UV-induced skin damage models, atopic dermatitis, psoriasis, fungal infections, and wound-healing assays. The biological effects were mainly associated with the reduction in reactive oxygen species (ROS), inhibition of pro-inflammatory cytokines such as TNF-α and IL-6, modulation of NF-κB and MAPK signaling pathways, suppression of matrix metalloproteinases, and improvement of skin barrier integrity and collagen synthesis. Overall, the findings highlight the therapeutic potential of phytocompounds and plant-derived extracts as promising agents for the prevention and treatment of various skin disorders. Polyphenols are generally the major class of phytocompounds with beneficial effects on the skin, causing anti-inflammatory action by decreasing the level of pro-inflammatory cytokines and interleukins (especially IL-4, IL-5 and IL-6) [1,8]. Also, in the case of applying extracts rich in polyphenols on atopic dermatitis, it can be confirmed that they determine a decrease in the level of cellular factor TNF-α, decrease the thickness of the epidermis, prevent the appearance of calluses, and decrease the level of free radicals, having an antioxidant and regenerative effect on the skin [100,101].

There are also other classes of phytocompounds presented in the specialized literature that are recognized for their beneficial, reparative, anti-inflammatory, anti-aging and senolytic properties, among which we can mention alkaloids, polysaccharides and even some vitamins found in plant extracts [102,103,104].

As a mechanism of action, alkaloids are notable for their ability to intervene in the attenuation of inflammatory processes through mechanisms similar to those of polyphenols, thus inhibiting the formation of pro-inflammatory factors and especially interleukins [105].

Alkaloids also have a strong antimicrobial capacity (preventing infection and superinfection of damaged skin) and the ability to stimulate the proliferation of specific cells such as fibroblasts and keratinocytes, accelerating the healing process [105,106].

The anti-aging activity of alkaloids is due to the ability of these phytochemicals to inhibit metalloproteinases and thus attenuate the destruction of collagen fibers [107,108].

Polysaccharides also intervene in the healing and treatment processes of dermatological lesions and multiple pathologies at this level. These phytochemical compounds act as skin regeneration stimulants, especially in the wound healing process. Polysaccharides found especially in *Aloe vera* or *Sansevieria trifasciata* extracts function as a natural dressing, favoring the absorption of wound exudates and accelerating the homeostasis and healing process [99,109]. They also have intrinsic antimicrobial properties, forming a physical barrier against pathogens and inhibiting the formation of bacterial biofilms [110]. The anti-inflammatory effect is attributed to their ability to form films and inhibit pro-inflammatory factors [99].

In plant extracts, in addition to the majority of phytochemical components, there are also minerals and vitamins whose properties have been highlighted in the recent specialized literature [12].

Thus, the presence of vitamins A, E or C, respectively, and minerals such as selenium, copper, and zinc also favors the process of maintaining skin homeostasis, healing and regeneration, especially through its demonstrated antioxidant capacity [104].

Vitamin A is responsible for the synthesis of epidermal proteins and cell proliferation, being directly involved in the process of the re-epithelialization and synthesis of collagen and elastin. Vitamin A actively participates in the angiogenesis process in the dermis, improving microcirculation. These processes directly influence the thickness of the skin, preventing thinning associated with aging or atrophic pathologies [104,111]. It is also a vitamin that is directly involved in reducing sebum production and limiting the proliferation of the Propionibacterium acnes bacteria, being an essential mechanism in the treatment of acne and seborrheic conditions [104,112].

Vitamin E plays an essential physiological role in maintaining skin integrity, actively participating in the biosynthesis of collagen, elastin and glycosaminoglycans (GAGs), fundamental elements for the elasticity and hydration of the dermal matrix [113]. In addition to structural support, it provides a critical defensive barrier by protecting the lipid structures of the stratum corneum and exhibits strong antioxidant properties, neutralizing the free radicals responsible for oxidative stress [104]. Moreover, the action of vitamin E is also photoprotective, demonstrated by the ability to prevent the development of erythema induced by UV radiation, reducing acute inflammation and premature cellular degradation at the level of skin tissue [104,114].

Vitamin C actively participates in the differentiation of keratinocytes and the stimulation of ceramide synthesis, strengthening the hydrolipidic barrier and optimizing skin hydration. At the level of the dermis, it enhances collagen biosynthesis and the formation of the extracellular matrix by increasing the synthesis of glycosaminoglycans (GAGs), thus ensuring the density and firmness of the tissue [115,116].

Selenium acts on the skin as a powerful antioxidant, reducing the level of free radicals, and also having an anti-aging effect, especially being photoprotective against UV radiation. Along with other minerals and vitamins, it stimulates the formation of elastin and collagen [117,118].

Copper is a key element in modulating melanin synthesis, but it is also recognized for its antimicrobial capacity along with phytocompounds existing in plants. Zinc is especially recognized for its ability to modulate the activity of 5α-reductase, a key enzyme in regulating sebum secretion, especially in conditions characterized by imbalances at this level such as acne [12].

## 6. New Strategies for Approaching Dermatological Treatments

In the field of dermatological pathologies, phytocompound-based therapies require a different approach in terms of the application of innovative phytopharmaceutical products to the skin. In other words, a strategic approach is needed to the treatments applied to different dermatological conditions, optimizing and increasing the efficiency of the applied treatment as much as possible.

At the skin level or by applying conventional pharmaceutical forms, there are several factors that can affect the release of substances of therapeutic interest from the pharmaceutical preparation, which leads to a decrease in the expected effect and to low absorption and bioavailability [15].

Among the physico-chemical factors that greatly influence the absorption of compounds of therapeutic interest through the skin, we can mention their solubility, molecular weight, and polarity. There are also physical and biological factors that affect bioavailability, including the area of application, skin permeability or existing conditions [119].

Taking into account these aspects listed above, new treatment strategies for dermatological conditions are aimed at formulations that are capable of releasing phytocompounds of therapeutic interest in a targeted, controlled and sustained manner. Such delivery systems are, for example, like those presented in Table 3, nano-systems such as liposomes, nanosomes, micelles, nanoemulsions, nanocolloids or nanocrystals that encapsulate the phytocompound of interest and release it in a controlled and targeted manner, thus considerably increasing the bioavailability of the treatment while avoiding the side effects often encountered in conventional dermatological treatments [120].

Table 3 summarizes the main innovative delivery systems that are currently employed for the incorporation of phytocompounds into dermatological therapies, highlighting their therapeutic roles and potential clinical applications. The data emphasize the growing scientific interest in advanced nanotechnological approaches designed to enhance the stability, bioavailability, and targeted delivery of bioactive compounds at the cutaneous level.

The delivery platforms presented include solid lipid nanoparticles, nanostructured lipid carriers, liposomes, nanoemulsions, nanoemulgels, exosomes, polymeric hydrogels, and green nanoparticles. These systems are specifically designed to overcome the limitations associated with conventional topical formulations, such as poor skin penetration, the reduced stability of phytocompounds, rapid degradation, and limited therapeutic efficacy. By encapsulating plant-derived bioactive compounds, these nanosystems facilitate controlled and sustained release, improve penetration through the stratum corneum, and maintain optimal concentrations of active substances at the target site.

The studies included in Table 3 demonstrate the broad applicability of these delivery systems in dermatological therapy. Lipid-based nanoparticles loaded with *Centella asiatica* were investigated for the management of scleroderma, while formulations containing *Elaeis guineensis*, *Mangifera indica*, or *Carthamus tinctorius* exhibited significant antioxidant and anti-aging activities [121,125,139]. Similarly, anti-inflammatory applications were reported for ginger extract, turmeric, and thymol incorporated into lipid nanoparticles or nanoemulgels, highlighting their potential in the treatment of inflammatory skin disorders such as acne vulgaris and irritant dermatitis.

Several delivery systems also showed promising applications in regenerative medicine and wound healing. For example, giant liposomes containing *Stellaria media*, as well as nanoemulsions formulated with *Tamarix aphylla* extracts, demonstrated regenerative, antioxidant, and wound-healing properties [132,137]. Moreover, exosome-based systems containing epigallocatechin gallate or *Physalis peruviana* extracts were associated with improved dermal fibroblast regeneration, tissue remodeling, and anti-psoriatic activity [135].

In addition, Table 3 highlights the increasing relevance of nanotechnology in skin cancer therapy and chemoprevention. Formulations containing *Withania somnifera*, chrysin, resveratrol, and polyphenol mixtures incorporated into nanostructured carriers exhibited anticancer, antioxidant, and chemoprotective effects, suggesting their potential as complementary therapeutic strategies in melanoma and other dermatological malignancies [8,93,100,107,126].

Overall, the findings presented in Table 3 support the concept that advanced delivery systems represent a major direction in contemporary dermatological research. The integration of phytocompounds into nanoscale carriers significantly enhances the therapeutic performance by improving the physicochemical stability, increasing dermal penetration, enabling a controlled release, and minimizing adverse reactions commonly associated with conventional therapies.

Consequently, these innovative systems offer promising perspectives for the development of safer, more effective, and targeted phytopharmaceutical formulations for dermatological applications.

## 7. Limitations and Future Perspectives

One of the main limitations of the current research field is related to the high variability associated with plant-derived extracts and phytocompounds used in dermatological applications. The biological activity of plant extracts is strongly influenced by multiple factors, including plant species, geographical origin, cultivation conditions, harvesting period, extraction methods, and storage conditions. As a result, significant differences in phytochemical composition may occur even between extracts obtained from the same plant species, which limits the reproducibility and comparability of the experimental results.

In addition, most studies investigating phytocompounds in dermatological disorders focus on isolated experimental models, predominantly *in vitro* assays or animal studies, while the clinical evidence in human subjects remains limited. Although numerous plant extracts demonstrate antioxidant, anti-inflammatory, antimicrobial, and regenerative properties, the translation of these findings into clinical practice is still insufficiently validated. Another important limitation is the lack of standardized protocols regarding extraction procedures, concentrations of active compounds, dosage regimens, and formulation strategies, which makes it difficult to establish clear therapeutic recommendations.

Furthermore, many phytocompounds exhibit poor physicochemical stability, limited skin penetration, and reduced bioavailability when applied through conventional topical formulations. Even though innovative delivery systems such as nanoparticles, liposomes, and nanoemulsions have shown promising results in improving therapeutic efficacy, their long-term safety, toxicity profile, and interactions with the skin microbiome are not yet fully understood.

Future perspectives in this field should therefore focus on the standardization of plant extracts and the identification of specific bioactive molecules that are responsible for therapeutic activity in dermatological conditions. More extensive clinical studies are required to validate the efficacy, safety, and long-term tolerability of phytocompound-based therapies in human patients. In addition, future research should investigate the synergistic effects between phytocompounds and advanced delivery systems in order to optimize skin penetration, controlled release, and targeted therapeutic action.

The integration of phytotherapy with nanotechnology, personalized medicine, and microbiome-oriented approaches may represent an important direction for the development of safer and more effective dermatological treatments. At the same time, interdisciplinary collaboration between pharmaceutical sciences, dermatology, biotechnology, and materials engineering will be essential for transforming experimental phytopharmaceutical formulations into clinically applicable therapeutic products.

## 8. Conclusions

Following the analysis of the specialized literature and the bibliometric study carried out, it is evident that skin health is a complex field, influenced by internal and external factors, such as UV radiation, oxidative stress, the skin microbiome and age-related physiological changes.

Common dermatological conditions, such as inflammation, photoaging, pigmentation disorders and skin lesions, are traditionally treated with synthetic substances. However, their use is limited by adverse effects and the emergence of therapeutic resistance.

In this context, phytotherapy becomes a promising alternative, thanks to bioactive compounds of plant origin (polyphenols, flavonoids, alkaloids, etc.), which exhibit antioxidant, anti-inflammatory, antimicrobial and regenerative properties. The integration of these compounds into modern delivery systems (nanoemulsions, liposomes, nanocarriers) contributes to the increasing bioavailability and therapeutic efficiency, while reducing side effects.

The bibliometric analysis confirms the increased interest of the scientific community in this field, highlighting the interdisciplinary nature of the research and international collaborations, including the active involvement of Romania.

In conclusion, the use of phytocompounds in the treatment of dermatological conditions represents a modern and effective direction, with significant development potential, although additional studies are necessary for the clinical validation and optimization of therapeutic formulations.

## Figures and Tables

**Table 1:** Examples of phytocompounds tested *in vitro* and *in vivo* and their mechanisms of action in the prevention and treatment of various skin conditions.

Phytocompounds	Test Type	Administration	Bioactivity	Mechanism of Action	Ref.
Dihydromyricetin	*In vivo* test on mice with induced psoriasis	Cream applied 1–6 days	Antipsoriasis, anti-inflammatory	↑ antioxidant activity; downregulated the expression of Ki67, IL-17, and IL-23 in mouse skin tissue; inhibited TNF-α-induced hyperproliferation of HaCaT cells.	[79]
Lupeol	*In vivo* and *in vitro* tests in models with atopic dermatitis induced	Oral	Anti-inflammatory against atopic dermatitis, antioxidant	↓ IgE and IgG2a; ↓ protein secretion of T helper 2 cytokines, Th1 cytokines, and pro-inflammatory cytokine; suppressed epidermal and dermal thickening.	[80]
Resveratrol	*In vivo* and *in vitro* tests	Different cosmetics formula	Anti-aging, antioxidant, photoprotective, anti-inflammatory	↓ ROS; inhibition of NF-κB and MMP expression; activation of SIRT1 signaling.	[81]
Epigallocatechin gallate (EGCG)	*In vivo* tests	Cream 2.5% 6 weeks	Anti-acne, anti-inflammatory, anti-angiogenic	↓ ROS; inhibition of inflammatory cytokines and NF-κB signaling; antiangiogenic activity; regulation of keratinocyte proliferation.	[82]
Thymol	*In vivo* and *in vitro* tests	Topical application (gel/nanoparticles)	Antimicrobial, antifungal anti-inflammatory	↓ inflammatory mediators and oxidative stress; antimicrobial and antioxidant effects.	[83]
Curcumin	*In vitro* and *in vivo* tests	Topical application; oral administration	Wound healing, anti-inflammatory	↓ ROS; inhibition of NF-κB and pro-inflammatory cytokines; promotion of collagen synthesis and wound repair.	[84,85]

↑—increase a parameter and ↓—decrease a parameter.

**Table 2:** Plant extracts and plant-derived materials tested *in vitro* and *in vivo* and their mechanism of action in the prevention and treatment of various skin conditions.

Plant Material/Plant Extract	Tests Type	Administration	Bioactivity	Mechanism of Action	Ref.
*Gaultheria procumbens* L.	*In vitro* on UVA-irradiated human dermal fibroblasts	Application of the extract in the range of 0.5–100 µg/mL	Anti-aging Antioxidant Anti-inflammatory	↑ Cell viability ↓ Kinase activity ↓ Inflammatory factors	[86]
*Green tea*	*In vitro* on dermal fibroblasts	Different concentration of the extract	Anti-aging Senolytic Senomorphic	↓ Inflammatory factors Antioxidant effect → ↓ ROS killing of cellular senescent cells without harming the “normal” cells	[87]
*Matricaria chamomilla*					
*Solidago virgaurea*					
*Reishi*					
*Lycium barbarum*					
*Acalypha indica*	*In vitro* tests	Extract application on fungal isolates from skin	Antifungal	Antifungal effect, MIC at 10–100 Ig/mL	[88]
*Lawsonia inermis*					
*Allium sativum*					
*Citrus limon*					
*Microsorum scolopendria*	*In vitro* on dermal fibroblast	Extract application	Antioxidant, Antifungal	↓ ROS Strong antifungal capacity against *S. aureus* and *S. epidermitis*	[89]
*Eisenia bicyclis* ethanolic extract	*In vitro* and *in vivo* tests	Application of the extract to keratinocyte cells and oral administration in mouse models	Photoprotective Anti-aging	Regulating MMP-1 and Procollagen type I production; Up-regulating glutathione peroxidase 1 (GPx1) and heme oxygenase-1 (HO-1);	[90]
*Centella asiatica*	*In vitro* on keratinocytes cell line	Extract application in range 100–500 µg/mL	Against atopic dermatitis Anti-inflammatory effect	Inhibited the expression of interleukin-6 (IL-6) and TNF-α	[90]
Cacao powder	*In vivo*	Oral	Anti-aging Anti-wrinkle	↓ UVB-induced wrinkles; regulation of genes involved in the production and maintenance of the dermal matrix; inhibition of UVB-induced matrix metalloproteinase-1 expression	[91]
*Crataegus pinnatifida* extract	*In vivo* on mice groups	Dermal application	Antioxidant Anti-inflammatory Antitumoral	↓ ROS ↓ edema ↓ the inflammatory process ↓ the incidence of tumor occurrence and ↓ the size	[92]
Grapefruit extract	*In vivo* on mice groups	Dermal application, cream with pectin–chitosan nanoparticles with extract included	Photoprotector against UV radiation Anti-aging	↓ eritema ↓ ROS ↓ skin peeling ↓ inflammation Down-regulation for biochemical markers associated with extracellular matrix degradation, inflammation, and basement membrane disruption.	[93]
*Anchusa azurea* methanolic extract	*In vivo* on mice with induced burn wounds	Dermal application, 1% and 10% ointment with plant extract	Wound healing Regenerator Antioxidant	↓ the inflammatory process ↓ IL-6 ↑ IL-10 ↑ the process of scarring and wound healing	[94]
*Melastoma malabathricum* ethanolic extract	*In vivo* on mice models	Dermal application 2.5%, 5%, 10% for 28 days	Nontoxic for the skin	Not significant modification for biochemical parameters → extract was non-toxic for the skin.	[95]
*Curcuma longa* and *Allium sativum* decoction	*In vivo* on rat models	Dermal application	Wound healing Antioxidant Antibacterial Anti-inflammatory	↓ ROS ↓ the circumference of the wound causes it to heal.	[96]
*Sigesbeckia pubescens*			Against atopic dermatitis	Inhibited proinflammatory chemokine production; Protect the skin barrier	[97]
*Black Soybean* Cultivar A63	*In vivo* on mice models with atopic dermatitis induced with oxazolone	Dermal application 22 days long	Against atopic dermatitis	↓ epidermal thickness ↓ inflammatory cell infiltration, ↓ Interleukin (IL)-4 and IL-5, restored damaged skin barrier tissues	[98]
*Sansevieria trifasciata*	*In vivo*	Application ointment with plant extract 5, 10, 20%	Reparative effect in callosities of toes	All participants in the study were improved within 4 weeks such that the recovery time for the 5% ointment was 25 days, while for 10% and 20% ointment, it was 15 and 10 days, respectively.	[99]

↑—increase a parameter and ↓—decrease a parameter.

**Table 3:** Phytocompounds included in various innovative delivery systems.

Plant Extract or Compound	Delivery System	Role	Ref.
*Centella asiatica*	Lipid nanoparticles	Against Sclerodermia	[121]
*Elaeis guineensis*	Solid lipid nanoparticles	Anti-aging	[122]
*Ginger extract*	Lipid nanoparticles	Anti-inflammatory	[123]
*Prunus persica* (L.) var. Florida Prince	Solid lipid nanoparticles	Skincare/cosmetics	[124]
*Mangifera indica* L.	Green nanoparticles	Oxidative stress and antiaging	[125]
Withania somnifera	Niosomes and solid lipid nanoparticles	Skin cancer/melanoma	[126]
Silybin extract	Solid lipid nanoparticles-enriched gel	Against irritant contact dermatitis	[127]
*Trans*-Resveratrol	Solid lipid nanoparticles	Skin disorder therapies	[128]
Mix of polyphenols	Nanostructured lipid carriers	Cancer prevention	[129]
*Carthamus tinctorius* L.	Solid lipid nanoparticle loaded polymeric hydrogels	Anti-aging	[130]
*Resveratrol*	Nanostructured lipid carriers and solid lipid nanoparticles	Antioxidant	[13]
*Eucalyptus or rosemary essential oils*	Solid lipid nanoparticles	Wound healing	[131]
*Stellaria media*	Giant liposome	Wound healing and antioxidant	[132]
*Turmeric*	Nanoparticle	Anti-inflammatory	[133]
*Epigallocatechin 3 gallate*	Exosome	Against psoriasis	[134]
*Physalis peruviana*	Exosomes and solid lipid nanoparticles	Dermal fibroblast regeneration and remodeling	[135]
*Thymol*	Nanoemulgels	Against acnea vulgaris	[14]
*Chrysin*	Nanoemulgel	Anticancer	[14]
*Essential oil*	Nanoemulsion	Antisepsis	[136]
*Tamarix aphylla*	Nanoemulsion	Regenerate burn wound healing	[137]
*Punica granatum*	Nanoemulgel	Potent anticancer, antimicrobial, and anti-inflammatory activities	[138]

## Data Availability

No new data were created or analyzed in this study. Data sharing is not applicable to this article.
